# Comparison Between Phenylephrine and Dopamine in Maintaining Cerebral Oxygen Saturation in Thoracic Surgery: A Randomized Controlled Trial

**DOI:** 10.1097/MD.0000000000002212

**Published:** 2015-12-11

**Authors:** Ji Won Choi, Hyun Joo Ahn, Mikyung Yang, Jie Ae Kim, Sangmin M. Lee, Jin Hee Ahn

**Affiliations:** From the Department of Anesthesiology and Pain Medicine, Samsung Medical Center, Sungkyunkwan University School of Medicine, 50 Ilwon-dong, Kangnam-gu, Seoul, Republic of Korea.

## Abstract

Fluid is usually restricted during thoracic surgery, and vasoactive agents are often administered to maintain blood pressure. One-lung ventilation (OLV) decreases arterial oxygenation; thus oxygen delivery to the brain can be decreased. In this study, we compared phenylephrine and dopamine with respect to maintaining cerebral oxygenation during OLV in major thoracic surgery.

Sixty-three patients undergoing lobectomies were randomly assigned to the dopamine (D) or phenylephrine (P) group. The patients’ mean arterial pressure was maintained within 20% of baseline by a continuous infusion of dopamine or phenylephrine. Maintenance fluid was kept at 5 mL/kg/h. The depth of anesthesia was maintained with desflurane 1MAC and remifentanil infusion under bispectral index guidance. Regional cerebral oxygen saturation (rScO_2_) and hemodynamic variables were recorded using near-infrared spectroscopy and esophageal cardiac Doppler.

The rScO_2_ was higher in the D group than the P group during OLV (OLV 60 min: 71 ± 6% vs 63 ± 12%; *P* = 0.03). The number of patients whose rScO_2_ dropped more than 20% from baseline was 0 and 6 in the D and P groups, respectively (*P* = 0.02). The D group showed higher cardiac output, but lower mean arterial pressure than the P group (4.7 ± 1.0 vs 3.9 ± 1.2 L/min; 76.7 ± 8.1 vs 84.5 ± 7.5 mm Hg; *P* = 0.02, *P* = 0.02). Among the variables, age, hemoglobin concentration, and cardiac output were associated with rScO_2_ by correlation analysis.

Dopamine was superior to phenylephrine in maintaining cerebral oxygenation during OLV in thoracic surgery.

## INTRODUCTION

A restrictive fluid regimen has been recommended in thoracic surgery because acute lung injury (ALI) is known to correlate with the amount of fluid administration during operations.^1^ One study suggests that, for every 500 mL increase in perioperative fluids, there is an odds ratio (OR) of 1.17 for developing ALI after lung resection.^2^ Slinger^3^ suggested that fluid should be restricted just to the point of maintaining a urine output of 0.5 mL/kg/h and vasoactive agents may be used if tissue perfusion is inadequate. Therefore, restriction of fluid administration and treatment of hypotension, which is not caused by major hemorrhage, with vasoactive agents, could be a basic concept in thoracic anesthesia.

Patients undergoing lung resection surgery usually receive one-lung ventilation (OLV). During OLV, a significant decrease in cerebral oxygenation can occur.^4,5^ Because the goal of hemodynamic optimization is to improve oxygen delivery to major organs, understanding how the administration of vasoactive agents affects cerebral perfusion and oxygenation, the most important organ in the body, would be of major clinical relevance, especially during OLV.

However, there are no data comparing vasoactive drugs in terms of maintaining cerebral oxygenation during thoracic surgery. Therefore, we compared 2 commonly used agents, phenylephrine and dopamine, continuous infusion with respect to maintaining regional cerebral oxygen saturation (rScO_2_) during OLV in major thoracic surgery.

Our hypothesis is that dopamine continuous infusion provides higher rScO_2_ during OLV than phenylephrine continuous infusion.

The primary aim of our study was to investigate the effects of phenylephrine and dopamine continuous infusion on rScO_2_ during OLV. As secondary outcome, we identified the hemodynamic variables which are responsible for the changes in rScO_2_ in major thoracic surgery.

## METHODS

This was a parallel-design, 1:1 allocation, randomized, double-blind (participants, assessors of outcomes) controlled study. The ClinicalTrials.gov ID is NCT02009007.

### Study Population

After approval by the Institutional Review Board of Samsung Medical Center, Seoul, Republic of Korea, 63 patients undergoing elective thoracic surgery for lung cancer in a tertiary care academic center, from January 2013 to December 2013, were recruited, and written informed consent was obtained from each patient. Eligibility criteria were: age >18 years, elective lobectomy or bilobectomy, American Society of Anesthesiologists (ASA) physical status I–III, and presenting with at least a 20% decrease in mean arterial pressure (MAP) during anesthesia. Exclusion criteria were: forced expiratory volume in 1 second <60% of predicted values, cerebrovascular disease, poorly controlled hypertension (systolic arterial pressure ≥160 mm Hg), poorly controlled diabetes mellitus (blood glucose ≥200 mg/dL), diuretics or antidepressant use before operation, and renal insufficiency (creatinine >1.5 mg/dL). The patients were randomly assigned to the dopamine (D) or phenylephrine (P) groups using the computerized random number generator program (www.randomizer.org). An enclosed assignment in a sequentially numbered, opaque, sealed envelope was allocated to each patient. This envelope was opened only by 1 author (HJA) at the theatre, and the card inside determined whether the patient was in the D or P group. The anesthesiologists who maintained blood pressure within 20% of baseline with the allocated drug were neither involved in data collection nor in interpretation of test results. Patients were also blinded to the allocation group.

### Anesthesia and Monitoring

After the patient's arrival in the operating theatre, a BIS monitor (Aspect Medical System, Norwood, MA) and 2 near-infrared spectroscopy (NIRS) probes (INVOS 5100C: Somanetics Corp., Troy, MI), 1 on each side of the forehead, were placed, in addition to the other routine monitors. After inducing anesthesia with fentanyl (1.5–2 μg/kg), propofol (2–3 mg/kg), and rocuronium (0.8 mg/kg), all patients were intubated with a double lumen endotracheal tube (Mallinckrodt, Covidien, Mansfield, MA) and maintained with balanced anesthesia using desflurance 1MAC and remifentanil (0.05–0.2 μg/kg/min). The depth of anesthesia was maintained similarly between BIS 40 and 55 in both groups. The remifentanil infusion rate was based on the patient's age, ASA physical status, and BIS monitoring. A radial arterial catheter and an esophageal Doppler probe (CardioQ, Deltex Medical, UK) were placed after tracheal intubation.

Baseline MAP was a value recorded when the patients arrived to the operating room and were stabilized for 10 minutes. The drug was started when the MAP decreased more than 20% of baseline after anesthesia. Dopamine was started at 5 μg/kg/min and phenylephrine at 1 μg/kg/min, and the infusion was adjusted for MAP to stay within 20% of baseline. SpO_2_ was maintained above 94% by adjustment of positive end-expiratory pressure (PEEP) and FiO_2_ during OLV.

The cerebral rScO_2_ was monitored by NIRS (INVOS 5100C: Somanetics Corp., Troy, MI) with optodes placed to the forehead above the frontal sinuses. INVOS 5100C employs a dual-detector system to subtract a shallow light path from a deep light path, allowing the derivation of the average oxyhemoglobin saturation in a volume of tissue about 2.5 to 3.0 cm below the skin. The device displays an approximation of venous-weighted hemoglobin saturation in tissue deep to the sensor.^6^ Mean values of right and left optodes were analyzed.

Cardiac output (CO) and stroke volume (SV) were monitored using an esophageal Doppler (CardioQ, Deltex Medical, UK). The esophageal Doppler measures blood flow velocity in the descending aorta. The aortic diameter is obtained from a built-in nomogram. SV is obtained by multiplying the blood flow distance and the cross-sectional area of the aorta.

Arterial blood gas was analyzed at baseline, 15 minutes after induction (TLVB), 30 and 60 minutes after OLV (OLV30, OLV60), and 15 minutes after the restart of two-lung ventilation (TLVA). All hemodynamic and rScO_2_ data were retrieved from the monitor after each case by the primary investigator who was not involved in anesthesia. The values of baseline, TLVB, OLV30, OLV60, and TLVA were recorded.

An intravenous infusion of lactated Ringer solution was maintained at 5 mL/kg/h throughout anesthesia. Blood loss was replaced by the same amount of hydroxyethyl starches. Packed red blood cell was transfused if the hemoglobin concentration fell below 8 g/dL. Pressure-controlled ventilation was used with a tidal volume of 6 mL/kg of predicted body weight and a ventilatory frequency of 8 to 12 bpm, with a target EtCO_2_ between 35 and 45 mm Hg. The arterial O_2_ saturation was monitored by a finger pulse oximeter (Nellcor, Covidien, Mansfield, MA).

### Postoperative Complications and Intensive Care Unit (ICU) Stay

Postoperative complications within 48 hours, the duration of mechanical ventilation, and ICU stay were compared between the 2 groups. Acute kidney injury (AKI) was measured by acute kidney injury network (AKIN) score. Postoperative delirium was monitored by the confusion assessment method in intensive care unit (CAM-ICU). Trained ICU nurses who were unaware of the study allocation assessed delirium every hour until ICU discharge.

### Statistical Analysis

Since no previous study exists, we referred a bolus study of similar vasoactives for sample size calculation, in which the mean difference of rScO_2_ was 10% between bolus phenylephrine and bolus ephedrine (*P* < 0.05).^7^ We regarded 15% as a clinically meaningful difference as a continuous infusion study. We conducted a pilot study to obtain standard deviations (SDs) of continuous infusions under OLV which were ±7% and ±9% in the D and P groups, respectively (n = 5, each group). We used 15% SDs for both groups as a more conservative approach. As a result, a sample size of 25 in each group was required with alpha level 0.05 and statistical power 80%. Considering a dropout rate of 20%, we chose a sample size of 30 per group. Sample size calculation was conducted by the software G∗Power 3.1.9.2.

The mixed-effect model was used to conduct within-group comparison across different time points. Between-group comparison was done at each time point by Student *t* test or Mann–Whitney *U* test, as appropriate. Bonferroni correction was used to adjust multiple comparisons. Normality test was done by Shapiro–Wilk test. Relationships between independent variables and rScO_2_ were tested using Pearson correlation analysis.

Results presented as mean ± SD for data normally distributed, and median (interquartile range [IQR]) for data not normally distributed and a value of *P* value 0.05 was considered statistically significant. Data were analyzed by the software program SAS 9.4 (SAS institute, Inc., Cary, NC).

## RESULTS

**FIGURE 1:**
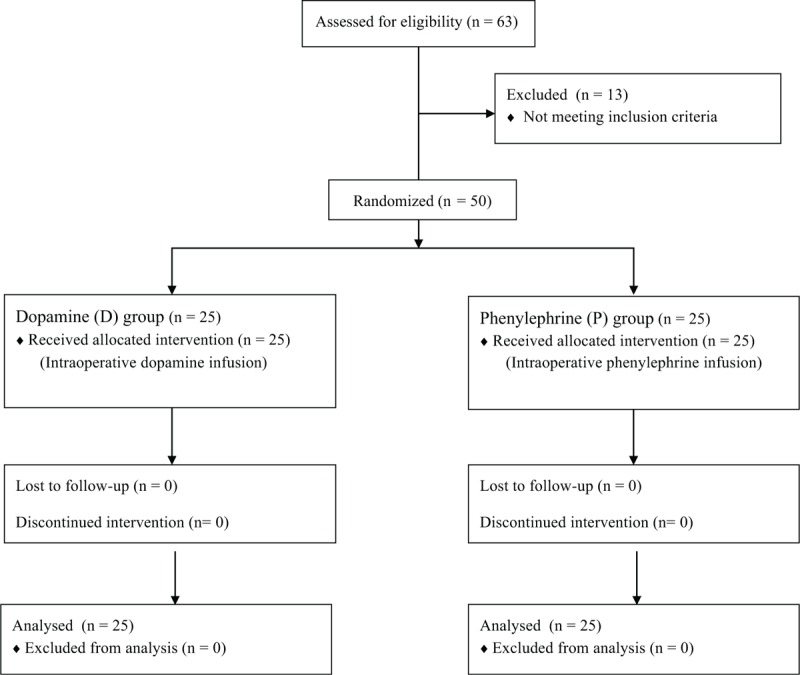
Flow diagram of patient selection.

Three patients refused enrollment. Ten patients did not develop a lower than 20% decrease in MAP after anesthesia and were excluded from the study. Fifty patients were able to finish the study (group D, n = 25; or group P, n = 25; Fig. 1). Detailed patients characteristics and planned surgeries are described in Tables 1 and 2. There were no differences in demographic and operative data between the 2 groups except urine output. It was higher in the D group than in the P group during operation (420 [282–720] vs 190 [90–340] ml; *P* < 0.001). Amount of anesthetics, fluid, blood loss, and mean hemoglobin concentration were not different between the groups. One patient of group D received a pack of red blood cell. SpO_2_ was maintained above 94% during OLV in all patients. Mean doses of dopamine and phenylephrine during OLV were 4.3 ± 1.9 and 0.8 ± 0.4 μg/kg/min, respectively. The rScO_2_, CO, HR, and MAP just before the drug infusion were not different between the D and P groups (rScO_2_, 73 ± 6% vs 72 ± 7%; CO, 4.2 ± 1.0 vs 3.9 ± 0.8 L/min; HR, 71 ± 12 vs 71 ± 13 bpm; MAP, 63 ± 6 vs 63 ± 6 mm Hg).

**TABLE 1:**
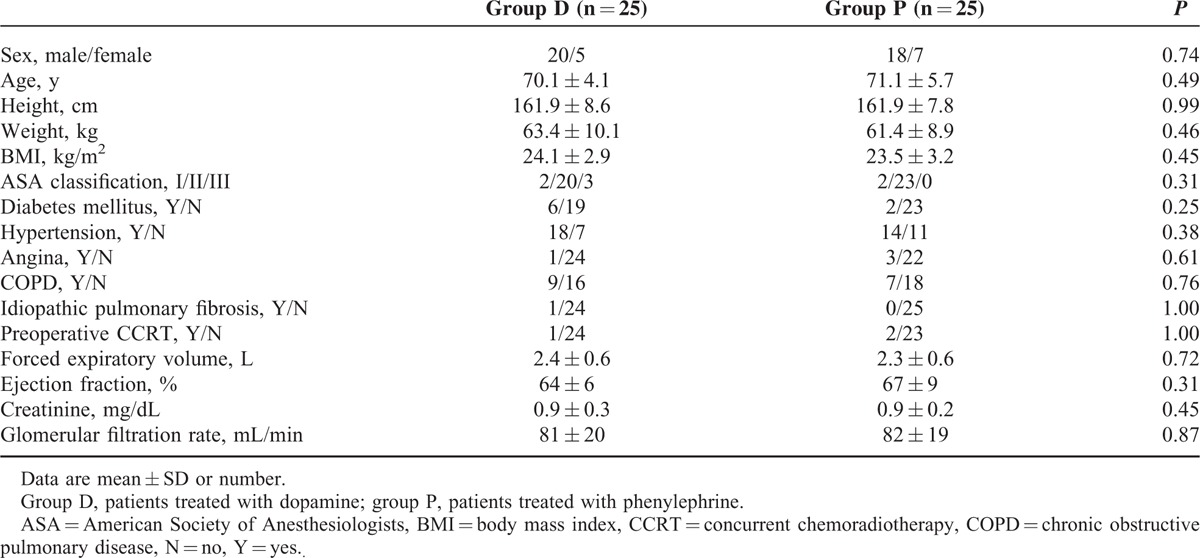
Patient Characteristics

**TABLE 2:**
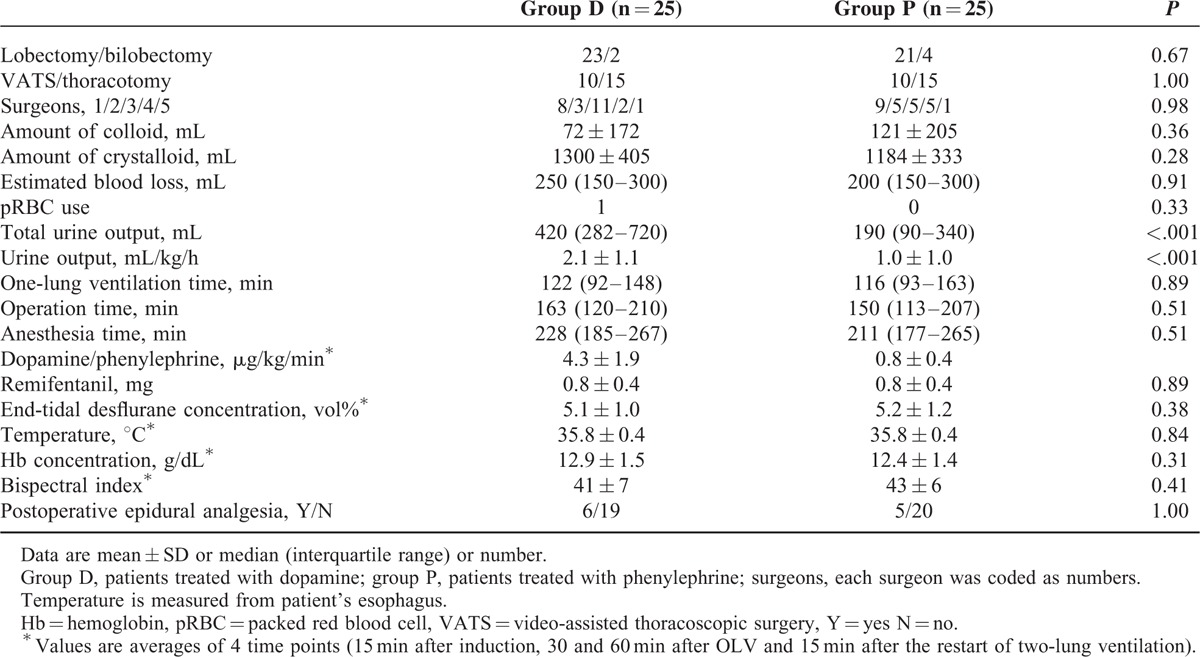
Operative Data

The D group maintained higher rScO_2_ than the P group during OLV (OLV30, 73 ± 6% vs 69 ± 9%, *P* = 0.12; OLV60, 71 ± 6% vs 63 ± 12%, *P* = 0.03; OLVmean, 72 ± 6% vs 66 ± 10%, *P* = 0.01; Table 3). The trend of rScO_2_ over time was different between the groups (*P* = 0.03) and within group D (*P* = 0.01) and P (*P* < 0.01). The rScO_2_ of baseline was higher than the rScO_2_ of OLV60 (*P* < 0.001) and TLVA (*P* = 0.02). The rScO_2_ of OLV30 was higher than the rScO_2_ of OLV60 (*P* < 0.001). In group D, the rScO_2_ of OLV30 was higher than that of OLV60 (*P* = 0.03). In group P, the rScO_2_ of baseline, OLV30, and TLVA were higher than that of OLV60 (*P* < 0.01, *P* < 0.01, *P* = 0.03, respectively). The number of patients whose rScO_2_ dropped >20% from baseline was 0 and 6 (24%) patients in the D and P groups, respectively (*P* = 0.02). The lowest rScO_2_ was 65 ± 6% and 60 ± 10% in the D and P groups, respectively (*P* = 0.02; Table 3).

**TABLE 3:**
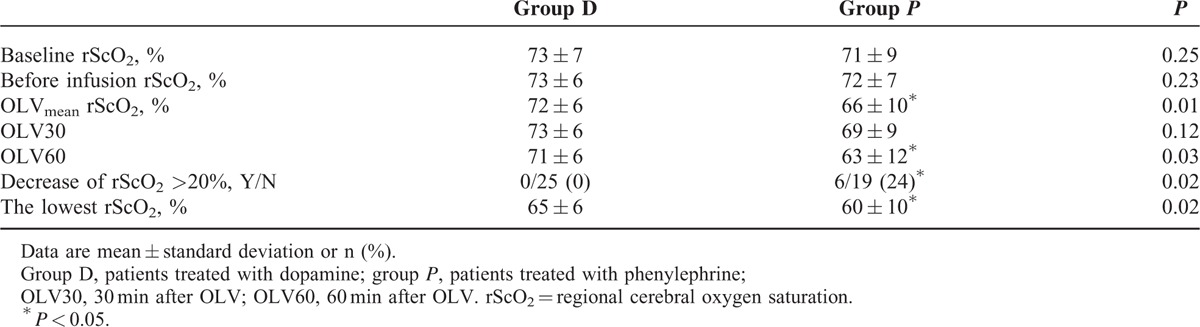
Regional Cerebral Oxygen Saturation (rScO_2_) During One-lung Ventilation

**FIGURE 2:**
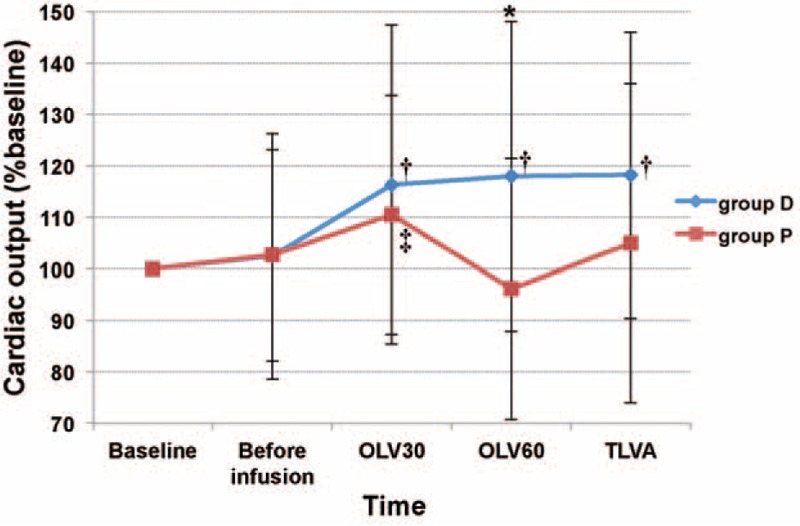
Group means and standard deviation for cardiac output during operation. Changes were depicted from a baseline set at 100%. ^∗^*P* < 0.05 between the 2 groups; ^†^*P* < 0.05 versus baseline of group D; ^‡^*P* < 0.05 versus baseline of group *P*. Before infusion, just before the drug infusion; OLV30, 30 min after one-lung ventilation; OLV60, 60 min after one-lung ventilaton; TLVA, 15 minutes after the restart of two-lung ventilation.

**FIGURE 3:**
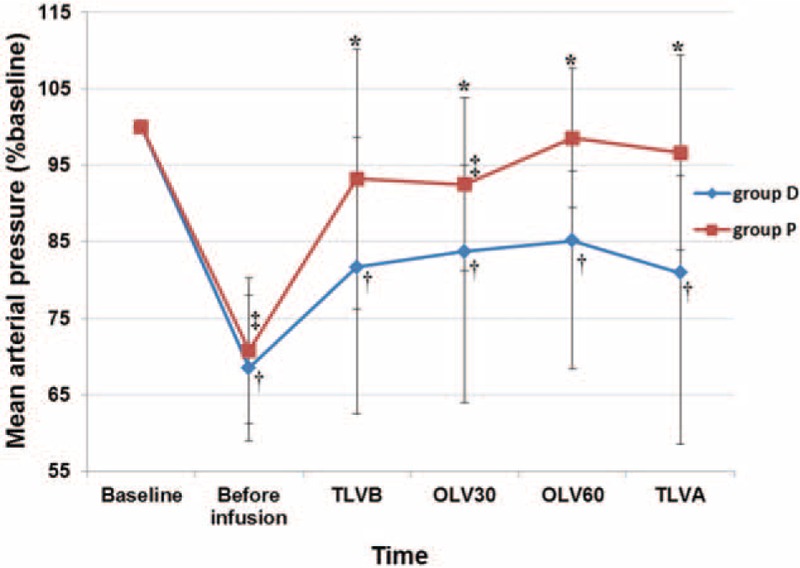
Group means and standard deviation for mean arterial pressure during operation. Changes were depicted from a baseline set at 100%. ^∗^*P* < 0.05 between the 2 groups; ^†^*P* < 0.05 versus baseline of group D; ^‡^*P* < 0.05 versus baseline of group *P*. Before infusion, just before the drug infusion; TLVB, 15 minutes after induction; OLV30, 30 min after one-lung ventilation; OLV60, 60 min after one-lung ventilation; TLVA, 15 minutes after the restart of two-lung ventilation.

The D group showed a higher CO and a lower MAP than the P group (CO, 4.7 ± 1.0 vs 3.9 ± 1.2 L/min; MAP, 77 ± 8 vs 85 ± 8 mm Hg; *P* = 0.02, *P* = 0.02, respectively; Table 4, Figs. 2 and 3). The CO of OLV30, OLV60, and TLVA were higher than that of baseline in the D group (*P* = 0.01, *P* < 0.01, *P* < 0.01, respectively). The CO of OLV30 was higher than that of baseline in the P group (*P* = 0.04) (Fig. 2). The MAP of TLVB, OLV30, OLV60, and TLVA were lower than that of baseline in the D group (*P* < 0.001, all). The MAP of OLV30 was lower than that of baseline in the P group (*P* < 0.01) (Fig. 3).

**TABLE 4:**
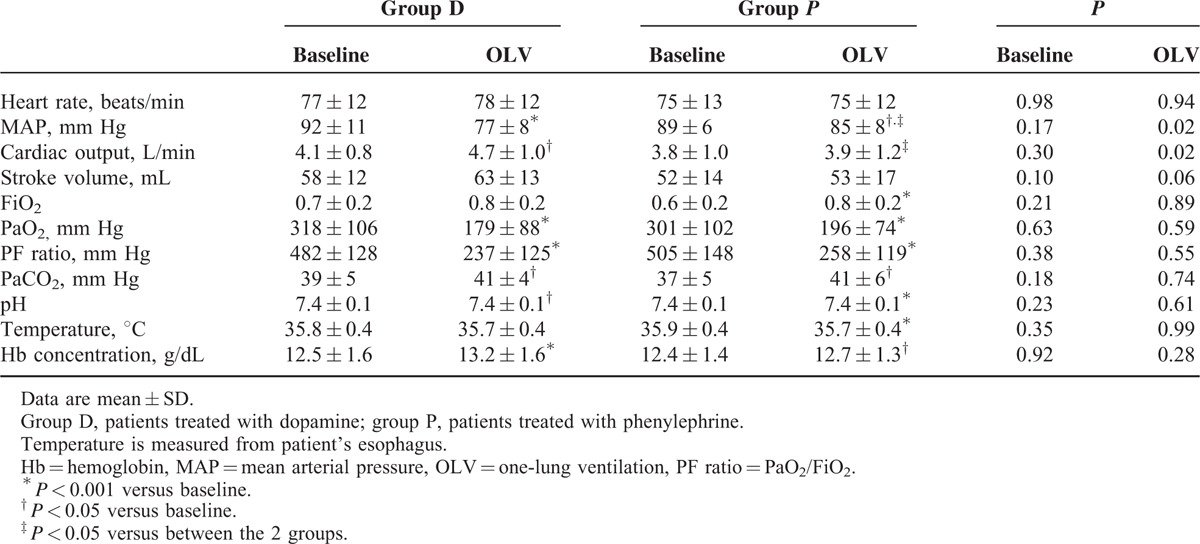
Hemodynamics and Blood Gas Analysis During One-lung Ventilation

Other variables, such as FiO_2_, PaO_2_, PaCO_2_, PaO_2_/FiO_2_ (PF ratio), pH, temperature, and hemoglobin concentration, were not different between the groups.

**FIGURE 4:**
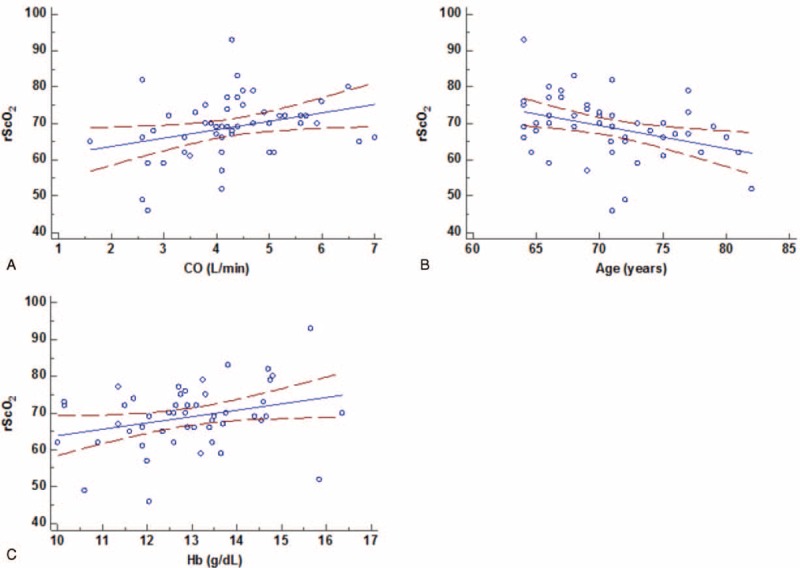
Factors related to rScO_2_. Pearson correlation analysis showed cardiac output (CO) (A), age (B), and hemoglobin concentration (Hb) (C) were associated with rScO_2_. Hb = hemoglobin, rScO_2_ = regional cerebral oxygen saturation.

Among variables, age, hemoglobin concentration, and CO were correlated with rScO_2_ (*r* = −0.37, 0.30, and 0.31; *P* = 0.01, 0.03, and 0.03, respectively) in the whole population. There was no correlation between rScO_2_ and other variables such as FiO_2_, PaO_2_, PaCO_2_, SpO_2_, PF ratio, pH, BIS, and OLV duration (Fig. 4) in the whole population.

There were no differences in the incidence of delirium (5 vs 6), AKI (2 vs 0), arrhythmia (4 vs 1), and mechanical ventilation (2 vs 3) between the D and P groups, respectively. There were no other serious respiratory or cardiac complications. Duration of ICU stay was similar (21.8 [18.3, 25.9] vs 21.7 [18.0, 24.6] h) between the D and P groups.

## DISCUSSION

Our study showed that dopamine is superior to phenylephrine in maintaining cerebral oxygen saturation for patients receiving OLV during thoracic surgery. Age, hemoglobin concentration, and CO were correlated with rScO_2._

The cerebral blood flow (CBF) decreases passively if the MAP falls below the lower limit of cerebral autoregulation, exposing the patient to possible cerebral hypoperfusion and ultimately, a reduction in rScO_2_. Immediate reductions in CBF and rScO_2_ have been reported when the MAP drops below 80 mm Hg.^8,9^ In our study, the MAP was higher in the P group than in the D group during OLV. However, the mean rScO_2_ was significantly lower in the P group than in the D group, and the difference of rScO_2_ between the 2 groups increased as OLV continued.

Only 3 studies^7,10,11^ have been published for the comparison of vasoactive drugs in relation to rScO_2_. In 1 study, patients received phenylephrine (100 μg intravenous [iv]) or ephedrine (10 mg iv) to restore MAP after anesthesia induction and rScO_2_ was monitored by INVOS cerebral oximeter. After administration of phenylephrine, MAP increased (51 to 81 mm Hg; *P* < 0.001), but rScO_2_ decreased by 14% (from 70% to 60%; *P* < 0.05). The administration of ephedrine led to a similar increase in MAP (53 to 79 mm Hg; *P* < 0.001) and preserved rScO_2_.^7^ Another study was a 2-treatment cross-over trial using Oxiplex TS cerebral oximeter; 1 bolus dose of phenylephrine (100–200 μg iv) and 1 bolus dose of ephedrine (5–20 mg iv). The rScO_2_ was significantly decreased after phenylephrine treatment (−3.2%; *P* < 0.01), but rScO_2_ was preserved after ephedrine treatment (+0.04%; *P* > 0.05).^10^ In a comparison study between phenylephrine and norepinephrine using FORE-SIGHT cerebral oximeter, phenylephrine and norepinephrine caused an equivalent increase in MAP [Δ = 13 [8–22] and Δ = 13 (9–19) mm Hg, respectively), and a similar decrease in cardiac index (both Δ = −0.2 ± 0.3 L/min/m^2^) and rScO_2_ [Δ = −4 [−7 to −2]% and Δ = −4 [−5 to −1)%, respectively).^11^

Phenylephrine and dopamine act in different mechanisms. Phenylephrine mainly increases vasomotor tone and dopamine mainly increases myocardial contractility and heart rate (HR). Vasoactive amines do not cross the blood–brain barrier; therefore direct action of either phenylephrine or dopamine on cerebral vessels would be negligible.^6^ Therefore, we speculate that the administration of phenylephrine may have reduced CO. Reduced CO by phenylephrine has been documented in both animals and humans.^12–14^ In animal experiments, phenylephrine increased arterial pressure by 30%, but decreased CO by 30%, ejection fraction by 36%, and SV by 25%.^14^ The CO, SV, and HR decreased significantly after phenylephrine treatment in anesthesia-induced hypotensive patients, whereas ephedrine preserved CO and SV, and increased HR.^10^ Phenylephrine leads to increased vagal tone as demonstrated by slowed HR, and this can also result in decrease in CO. A significant linear relationship was shown between CO and the middle cerebral artery mean flow velocity.^15,16^

Second, phenylephrine may constrict cerebral vessels indirectly through reflexive sympathetic nerve activation. Cerebral arteries are innervated by sympathetic nerve fibers abundantly,^17,18^originating from the superior cervical ganglion.^19^ Activity of sympathetic nerves on cerebral vessels increases promptly after a phenylephrine-induced rapid increase in arterial pressure.^20^ In anesthetized patients, the phenylephrine bolus injection increased the MAP by 20% to 30% and caused a significant decrease in CBF.^12^

In the current study, age, hemoglobin (Hb) concentration, and CO, but not MAP, were identified as the variables associated with rScO_2_. Age and Hb concentration were not different between the D and P groups. Therefore, the distinctive effect of phenylephrine and dopamine on rScO_2_ may be mainly explained by their different impacts on CO. In the study by Meng et al, which compared phenylephrine and ephedrine boluses,^10^ CO was identified to have the most significant association with rScO_2_, and after taking CO into consideration, the other physiological variables were not significantly associated with rScO_2_. Therefore, it seems that higher CO is the key factor of higher rScO_2_ shown in the D group, compared with the P group. Other variables which can possibly influence rScO_2_ such as FiO_2_, SpO_2_, PaO_2_, PaCO_2_, pH, temperature, and depth of anesthesia, were controlled during OLV in this study, and there were no differences between the groups.

Low rScO_2_ measured by NIRS was associated with an increase of neurological damage, cognitive impairment, or postoperative complications during cardiac and thoracic surgery.^5,21,22^ However, there is no proven cut-off value of rScO_2_ below which cerebral ischemia may occur because there is substantial inter and intrapatient variability in rScO_2_.^23^ In our study, incidence of delirium defined by CAM-ICU was not different between the 2 groups, and there were no postoperative cerebrovascular incidents. Urine output was higher in the D group during operation, but the incidence of AKI was not different between the 2 groups. Incidence of new-onset arrhythmia or ICU stay was not different either. However, our study was not powered for complications. Further studies focused on clinical outcomes are required.

For limitation, this study was a monocentric approach of hemodynamic control to compare inotrope and vasopressor; therefore, it does not exactly reflect clinical practice which employs multimodal approach for hemodynamic control. Inotropes including dopamine have several side effects, such as increasing oxygen consumption of the heart muscle and leading to tachycardia, arrhythmia, and a higher risk for cardiac events.^24,25^ Therefore, risk–benefit balance should be taken into consideration, and individualized approach is required for each patient.

Secondly, we did not measure CBF directly, but rather oxygenation of the brain. Usually, changes in rScO_2_ parallel those in middle cerebral artery flow velocity (MCAV).^8,26,27^ An important role of CBF is to provide adequate oxygenation for the brain; therefore monitoring rScO_2_ instead of middle cerebral artery flow may be more relevant.

Thirdly, we measured CO using an esophageal Doppler. Vasoactive drugs could affect the diameter of the aorta,^28^ thus making esophageal Doppler measurement less accurate. However, esophageal Doppler is less invasive than pulmonary artery catheter and more accurate than most of the noninvasive CO monitors under the influence of vasopressors.^29–31^

Fourthly, phenylephrine may constrict scalp vessels and result in the reduction of rScO_2_.^32^ INVOS 5100C is supposed to measure rScO_2_ of 2.5 to 3.0 cm below the skin. However, the effect of scalp vessel constriction cannot be absolutely ruled out.

Final limitation is the lack of a control group. However, a control group that would not receive any treatment when blood pressure decreases was considered unethical. In previous reports, rScO_2_ dropped significantly when no drug intervention was given to treat hypotension during OLV.^4,5^

In summary, cerebral oxygenation decreases after phenylephrine administration, but is maintained after dopamine continuous infusion in thoracic surgery patients undergoing OLV. The higher CO shown with the dopamine infusion may be beneficial in terms of maintaining cerebral oxygenation during OLV. We suggest that the increase in MAP is not a prerequisite to secure cerebral oxygenation, and CO should be increased to keep the optimum range of cerebral oxygenation.
