# Comparison Between Phenylephrine and Dopamine in Maintaining Cerebral Oxygen Saturation in Thoracic Surgery: A Randomized Controlled Trial: Erratum

**DOI:** 10.1097/01.md.0000480961.17450.a6

**Published:** 2016-02-12

In the article “Comparison Between Phenylephrine and Dopamine in Maintaining Cerebral Oxygen Saturation in Thoracic Surgery: A Randomized Controlled Trial”,^1^ which appeared in Volume 94, Issue 49 of *Medicine*, Dr. Hyun Joo Ahn's name was spelled incorrectly in the original article. The article has since been corrected online.
